# The *CTDP1* Founder Variant in CCFDN: Insights into Pathogenesis, Phenotypic Spectrum and Therapeutic Approaches

**DOI:** 10.3390/ijms27010034

**Published:** 2025-12-19

**Authors:** Iulia Maria Sabau, Alexandra Chera, Victor Gabriel Ungureanu, Mircea Cretu Stancu, Adela Chirita-Emandi, Matthew Wood, Maria Puiu, Octavian Bucur

**Affiliations:** 1 Doctoral School, Victor Babes University of Medicine and Pharmacy, 300041 Timisoara, Romania; iulia-maria.sabau@umft.ro (I.M.S.); maria.puiu@genomica.gov.ro (M.P.); 2 Genomics Research and Development Institute, 020021 Bucharest, Romania; 3 Doctoral School, Carol Davila University of Medicine and Pharmacy, 050474 Bucharest, Romania; 4 Institute of Biochemistry of the Romanian Academy, 060031 Bucharest, Romania; 5 Genomics Discipline, Carol Davila University of Medicine and Pharmacy, 050474 Bucharest, Romania; 6 Department of Microscopic Morphology Genetics Discipline, Center of Genomic Medicine, Victor Babes University of Medicine and Pharmacy Timisoara, 300041 Timisoara, Romania; 7 Regional Center of Medical Genetics Timis, Emergency Clinical Hospital for Children “Louis Turcanu”, 300011 Timisoara, Romania; 8 Medical Sciences Division, Oxford Harrington Rare Disease Centre, Department of Paediatrics, University of Oxford, Oxford OX3 7TY, UK; matthew.wood@paediatrics.ox.ac.uk; 9 Viron Molecular Medicine Institute, Boston, MA 02108, USA

**Keywords:** CCFDN, *CTDP1*, aberrant splicing, Roma population, founder variant, congenital cataract, facial dysmorphism, neuropathy

## Abstract

Congenital Cataracts, Facial Dysmorphism, and Neuropathy (CCFDN) syndrome is a rare autosomal recessive disorder predominantly found among Vlax Roma populations, caused by a deep intronic founder variant in the *CTDP1* gene. This review synthesizes recent advances in understanding the molecular mechanisms of *CTDP1* dysfunction, highlighting its central role in transcriptional regulation, RNA splicing, DNA repair, and genome integrity. The unique splicing defect caused by the founder disease-causing variant in the Roma population results in a multisystem phenotype with early-onset neuropathy, congenital cataracts, and characteristic facial dysmorphism. Beyond its genetic homogeneity, CCFDN displays variable clinical severity and presents diagnostic challenges due to overlapping syndromic features. We discuss the emerging therapeutic landscape, focusing on antisense oligonucleotides, small molecule modulators, gene replacement, and genome or transcriptome editing strategies, while emphasizing the challenges in targeted delivery and efficacy. Ongoing insights into *CTDP1*’s broader biological functions and population genetics inform new directions for diagnosis, genetic counselling, and the development of effective therapies for this severe yet underrecognized disorder.

## 1. Introduction

Congenital Cataracts, Facial Dysmorphism, and Neuropathy (CCFDN) Syndrome is a rare autosomal recessive disorder caused by the deep intronic variant c.863+389C>T (or IVS6+389C>T) in the Carboxy-Terminal Domain phosphatase subunit 1 (*CTDP1*) gene. This disease-causing variant is responsible for an aberrant splicing process involving the activation of an upstream cryptic splice acceptor site, which leads to the inclusion of an Alu sequence of 95 nucleotides in the processed *CTDP1* mRNA, ultimately resulting in a premature termination signal. Therefore, unless the mutant transcript is discarded through nonsense-mediated decay, the process of protein synthesis is altered, resulting in faulty, non-functional proteins [1,2,3]. The early-onset nature of symptoms, progressive neuropathy, and lack of curative treatments underscore the urgent need for a better understanding of the molecular pathology of CCFDN.

Although the condition was initially discovered in patients of Roma ethnicity from Bulgaria, cases have subsequently been documented in various other countries. The *CTDP1* disease-causing variant responsible for CCFDN is unique to the Vlax Roma subgroup, particularly prevalent among the Rudari, with an estimated carrier rate of 7%, while lower carrier rates (~1%) have been observed within other Roma subgroups [4,5]. Currently, over 190 individuals have been diagnosed with this rare condition, all sharing Roma ancestry, according to a publication updated in 2022. However, the actual prevalence of CCFDN remains unknown, and is probably underestimated because of underdiagnosis [1].

## 2. Genetic Basis and Molecular Mechanism

### 2.1. Structure and Function of CTDP1

CTDP1 encodes the eponymous protein known as Carboxy-Terminal Domain (CTD) phosphatase subunit 1, which it is also referred to as F-Cell Production 1 (FCP1), or Transcription factor IIF—associating CTD phosphatase, reflecting its association with the RAP74 subunit of transcription factor IIF. Other synonyms include CTD phosphatase subunit 1, RNA polymerase II subunit A CTD phosphatase, and serine phosphatase FCP1a [5]. These alternative names highlight the enzymatic role of CTDP1 as a phosphatase, which is able to dephosphorylate the CTD of POLR2A, the largest subunit of RNA polymerase II, thereby regulating transcription and gene expression [6].

**Figure 1:**
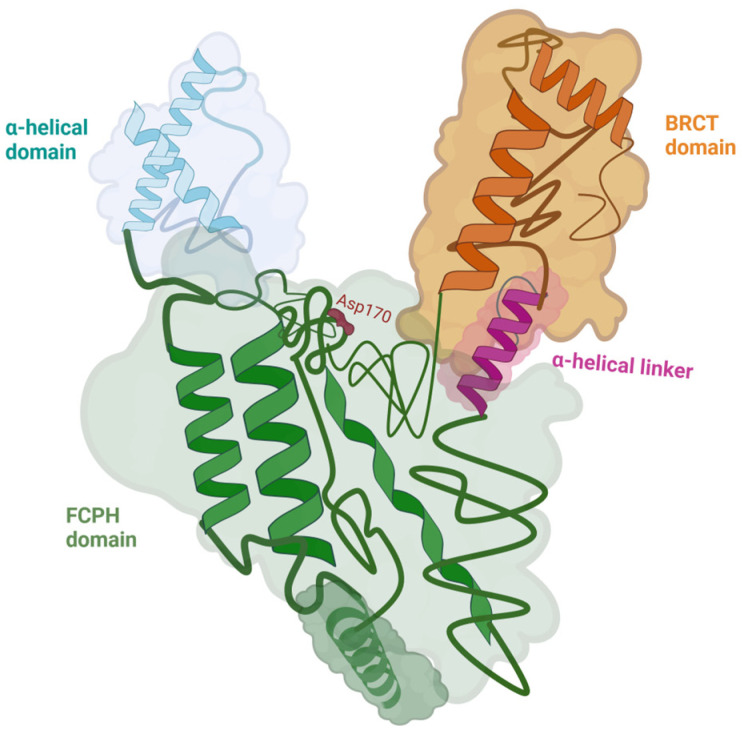
The structure of *CTDP1* (aa 140–580) with its domains: the FCPH domain colored in green, the BRCT domain colored in orange, and the α-helical domain colored in light blue. The FCPH and BRCT domains are connected by an α-helical linker colored in fuchsia. Asp170, which is a part of the active site within the FCPH domain, is represented in red. The CTDP1 amino acids 140–580 capture the enzymatic activity of the protein [7,8]. Created in BioRender. Chera, A. (2025) https://BioRender.com/yqpgb5e (accessed on 15 December 2025).

The protein is characterized by a multi-domain, Y-shaped structure with distinct regions that provide its enzymatic and regulatory properties. The 3 main domains of CTDP1 are: the FCP homology (FCPH) domain (colored in green), the α-helical domain (colored in light blue), and the C-terminal BRCT (BRCA1 C-terminal) domain (colored in orange) (see Figure 1). Notably, the study did not examine the full-length protein but instead focused on truncated constructs, as specific structural segments were sufficient to retain full enzymatic activity [7]. The core catalytic region is the FCPH domain, which contains the conserved DxDxT motif responsible for the phosphatase activity. This domain belongs to the haloacid dehalogenase (HAD) superfamily and is responsible for the Y-shaped architecture. The FCPH domain and surrounding structural components form a deep canyon that contains the active site, offering a precise binding pocket for the CTD of RNA polymerase II, the main substrate of CTDP1. The FCPH and BRCT domains are connected by an α-helical linker (colored in fuchsia in Figure 1), which contributes to the structural stability and proper spatial arrangement of these functional regions [7]. The BRCT domain distinguishes CTDP1 from other CTD phosphatases, while also mediating critical protein–protein interactions, particularly in the DNA damage response. This BRCT domain facilitates CTDP1’s interaction with key DNA repair proteins, including those involved in the pathway of Fanconi anemia, thereby playing an essential role in maintaining genome integrity and coordinating transcription with DNA repair processes [8].

Since 1998, CTDP1 phosphatase has been recognized as a central regulator of RNA polymerase II (Pol II) activity by catalyzing the removal of phosphate groups from its C-terminal domain (CTD) [9,10]. RAP74 is a core subunit of Transcription Factor IIF (TFIIF), a general transcription factor that associates with RNA polymerase II (Pol II) during transcription initiation and elongation. RAP74plays a key role in recruiting Pol II to promoters and stabilizing the transcription initiation complex. RAP74 also modulates transcription elongation by regulating Pol II processivity and assisting in promoter clearance [11]. Notably, RAP74 interacts with CTDP1, facilitating the targeted dephosphorylation of Pol II’s C-terminal domain (CTD), which is crucial for recycling Pol II back to its hypophosphorylated initiation-competent form [9].

Recent insights have uncovered the molecular interplay between CTDP1 and RPB7, a polymerase II subunit. RPB7 is one of the 12 subunits composing RNA polymerase II, specifically consisting of the ‘stalk’-like structure of the polymerase, which is involved in communication between Pol II and other transcription machinery components and RNA processing factors. RPB7 has structural and regulatory roles, acting as a scaffold to recruit factors necessary for efficient transcription and RNA processing [12]. Zheng et al. showed that RPB7 facilitates the recruitment of CTDP1 to the polymerase II complex, where CTDP1 is able to dephosphorylate the hyperphosphorylated CTD and the linker regions of RPB1. This enzymatic activity is critical because the unmodified hyperphosphorylated polymerase II can become a substrate for the E3 ubiquitin ligase Cullin 3 (CUL3), which marks it for proteasomal degradation. Thus, CTDP1 prevents RPB1 from ubiquitin-mediated proteolysis, ensuring that the polymerase remains available for new transcription cycles [13]. This novel discovery highlights the role of CTDP1 in connecting the stability of polymerase II with transcriptional reinitiation and RNA processing, adding a new dimension to the understanding of gene expression regulation.

**Figure 2:**
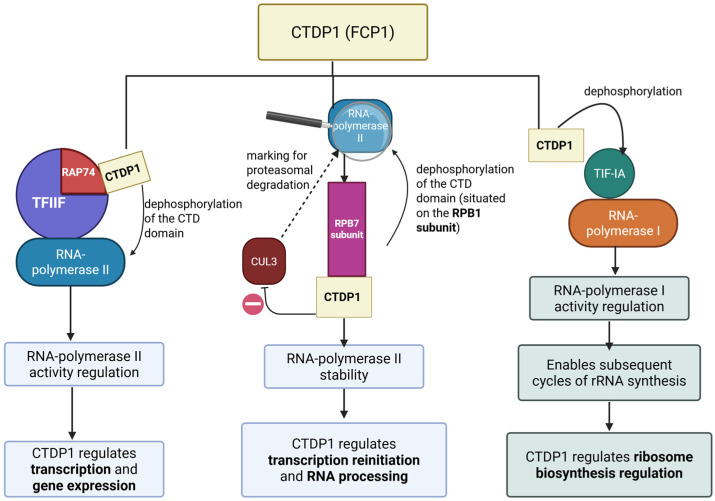
The effects of CTDP1 on regulating the activity of the RNA polymerases (I, II). Created in BioRender. Chera, A. (2025) https://BioRender.com/meb953z (accessed on 15 December 2025).

While, on one hand, the role of CTDP1 in transcription through modulating the activity of RNA polymerase II is well established, its regulatory influence extends to RNA Polymerase I, the enzyme responsible for synthesizing ribosomal RNA (rRNA). Specifically, CTDP1 dephosphorylates the transcription initiation factor (TIF-IA) localized at serines 170 and 172. This dephosphorylation step is necessary after the phosphorylation of TIF-IA through casein kinase 2 (CK2), an event that temporarily releases TIF-IA from the polymerase I complex so that transcription can proceed. By removing these phosphate groups, CTDP1 enables TIF-IA to reassociate with the polymerase machinery, thus enabling subsequent cycles of synthesis of rRNA. The disruption of this regulatory mechanism prevents the reassociation of TIF-IA with RNA polymerase I, thereby blocking subsequent rounds of rRNA transcription and compromising ribosome biogenesis. These findings underscore the important role of CTDP1 in ribosome biosynthesis regulation and cell proliferation [14] (see Figure 2).

On the other hand, Licciardo et al. explored the protein interactions of CTDP1 in a lung cancer cell line and discovered that it interacts with both RNA polymerase II and MEP50. The latter is a protein involved in the assembly of spliceosomal small nuclear ribonucleoproteins (snRNPs), as well as in methylation. Researchers found that *CTDP1* can interact with specific components of the pre-mRNA spliceosomal snRNPs, including the SmB protein, U1 SnRNP 70 protein, and U1 snRNA, with this interaction being mediated through MEP50 [15], therefore *CTDP1* may be considered as a bridge between transcription and RNA splicing, coordinating transcription elongation with the splicing machinery, in order to ensure proper transcript processing.

### 2.2. Functional Studies Reveal Additional Roles of CTDP1 Gene

**Figure 3:**
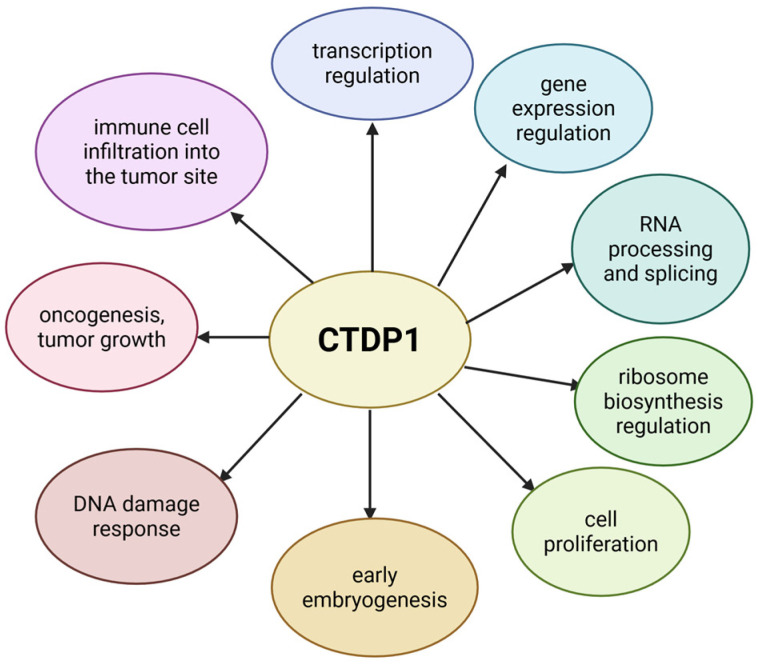
The roles of *CTDP1.* Created in BioRender. Chera, A. (2025) https://BioRender.com/739xspn (accessed on 15 December 2025).

Alongside its essential roles in cell proliferation, gene expression regulation, transcription and RNA splicing, many other functions have been attributed to *CTDP1* through functional studies (see Figure 3).

In 2021, Qiao et al. [16] investigated the function of *CTDP1* in the mammalian system, using a conditional knockout mouse model. By conducting this study, they have uncovered an indispensable role of this enzyme in early embryogenesis. Biallelic deletion of *CTDP1* has caused embryonic death before day 7.5, which was illustrated by widespread cell death and resorption of the embryo. *CTDP1* knockout in mouse embryonic fibroblasts (MEFs) triggered cell cycle arrest, with an accumulation of cells in G1 and G2/M phases and a reduction in cells found in the S-phase. These cell cycle abnormalities brought on by the deletion of *CTDP1* in murine models are also linked to a decrease in the level of phosphorylated RB, phosphorylated histone H3 and cyclin B, and an increase in the expression of the p27 protein. These findings suggest that the phosphatase activity of *CTDP1* is critical for transcription and for cell cycle progression, potentially through post-transcriptional regulation of checkpoint proteins. Mice with a heterozygous *CTDP1* genotype have lower levels of the homonymous protein (~40–50% compared to baseline) in various tissues (brain, lung, spleen, and heart) [16], without exhibiting any CCFDN-like phenotype. This supports the study conducted by Varon et al., showing that the threshold for observing CCFDN-like phenotypes is ~30% of functional *CTDP1* protein expression [2], highlighting the critical role of *CTDP1* expression levels in maintaining proper gene activity and cell growth, especially in tissues affected by CCFDN, like nerve cells and Schwann cells.

Studies have shown that *CTDP1* has important functions in DNA damage response. CTDP1 is the only phosphatase in the human proteome that also includes a BRCT domain [8], suggesting that it may have a special function in controlling the phosphorylation-mediated signalling that participates in the DNA damage repair [17]. Hu et al. demonstrated that *CTDP1* can directly interact with key proteins of the Fanconi anemia DNA repair pathway, including *FANCA, FANCI*, and *FANCD2*, which are known to participate in the repair of interstrand DNA crosslinks [8]. Their study revealed that *CTDP1* regulates the activation of FANCI through phosphorylation, chromatin localization, and recruitment to DNA damage sites. Furthermore, *CTDP1* expression enhances the efficiency of homologous recombination repair. Knockdown of *CTDP1* has increased the sensitivity of the cells to DNA interstrand crosslinks and double-strand breaks. In addition, *CTDP*1 knockdown interfered with the development of breast cancer cell lines, both in vitro and in vivo [8], underscoring the roles of *CTDP1* as a regulator of genome stability and revealing its potential as a therapeutic target in cancer or diseases with impaired DNA damage repair.

Other studies investigated the role of *CTDP1* in cancer and oncogenesis, for example through exposing gastric cancer cells to lentivirus-mediated small interfering RNA (siRNA) in order to silence *CTDP1* expression. The inhibition of *CTDP1* led to lower rates of proliferation in neoplastic cells, interruption of cell cycle at G0/G1 phase, as well as an enhanced apoptosis of gastric cancer cells. Moreover, *CTDP1* knockdown has also inhibited the capacity of gastric cancer cells to form colonies [18]. When combined, these findings show that *CTDP1* plays a major role in tumor growth, making it a promising therapeutic target for the treatment of gastric cancer. Similarly, very recent research has further highlighted *CTDP1*’s oncogenic potential. Through a study on ovarian cancer cell lines, Ning et al. revealed that high expression levels of AQP5 and *CTDP1* were strongly associated with immune cell infiltration [19]. The analysis of a gene regulatory network focused on the immune microenvironment showed a positive correlation between AQP5/CTDP1 and immune cell infiltration into the tumor site. A risk model based on Cox and LASSO regression analyses was made, showing that both AQP5 and *CTDP1* have a significant impact on the prognosis of cancer patients, indicating that they may be used as prognostic markers [19].

### 2.3. Expanding Role of CTDP1 as an Autoimmune Biomarker in Human Disease

In addition to its well-characterized role in transcriptional regulation, *CTDP1* has been implicated in genetic and autoimmune pathologies. A proteome-wide autoantibody screening has identified *CTDP1* as a novel autoantigen highly specific to Behcet disease, an autoimmune systemic vasculitis, highlighting *CTDP1*’s multifaceted biological roles beyond inherited disorders. Anti-CTDP1 autoantibodies exhibit high specificity for Behcet disease diagnosis and may influence tissues with rapid metabolism such as mucosa and skin [20]. Together, these findings underscore the critical importance of *CTDP1* in human health and disease, from transcriptional regulation and genetic syndromes to autoimmune mechanisms, and support its relevance for diagnostic and therapeutic strategies in diverse clinical contexts.

## 3. *CTDP1* Intronic Variant: Mechanism and Founder Origin

Congenital Cataracts, Facial Dysmorphism, and Neuropathy (CCFDN) is a rare disorder caused by pathogenic or likely pathogenic genetic variants of the *CTDP1* gene. Currently, the only pathogenic variant that has been described is the Romani founder intronic variant c.863+389C>T [1]. Therefore, CCFDN syndrome can be considered a genetically homogeneous condition, due to the fact that all patients are homozygous for the same ancestral disease-causing variant in the *CTDP1* gene.

The aforementioned intronic variant, present in the homozygous state, leads to aberrant splicing by activating a cryptic splice acceptor site located upstream of the mutation. The C→T substitution at position IVS6+389 creates a perfect donor splice site (gt) that activates an upstream cryptic acceptor site within the antisense Alu element, resulting in the inclusion of exactly 95 nucleotides of Alu sequence between the newly created donor site and the upstream acceptor site [2]. Alu elements are the most abundant short interspersed nuclear elements (SINEs) in the human genome, comprising over 10% of the genome sequence. These primate-specific retrotransposons are able to influence gene expression and genome stability by affecting splicing, transcriptional regulation, and recombination [21]. In the case of CCFDN, the aberrant splicing is driven by the strong de novo donor splice site created by the C→T mutation, which couples with a pre-existing cryptic acceptor site located 95 nucleotides upstream, causing exonization of the intervening intronic sequence. While this sequence happens to be derived from an Alu element, the splicing defect is caused by the point mutation creating the donor site, not by the Alu sequence itself. The altered transcript introduces a premature stop codon 17 amino acids downstream of exon 6, resulting either in triggering nonsense-mediated decay or in generating a truncated protein lacking the nuclear localization signal [22].

The splicing impact of *CTDP1* founder variant is partial rather than complete. RT-PCR analysis using competitive allele-specific amplification demonstrated the presence of both wild-type (193 bp) and aberrant transcripts (288 bp containing the 95-nucleotide inclusion) in all cell types from CCFDN patients, including fibroblasts, lymphoblastoid cells, myoblasts, and Schwann cells [2]. Real-time PCR quantification revealed that wild-type transcript levels were 15–35% of control levels, with variation related to cell type and culture conditions. The homozygous nature of all patients simplifies this quantification, as both alleles carry the same mutation, allowing direct measurement of the ratio between normal and aberrant splicing products. According to Qiao et al. the partial splicing defect results in approximately 30% functional CTDP1 protein and 70% truncated, non-functional protein [16]. This unique splicing defect is the first example of a transcriptional syndrome caused by the disruption of RNA polymerase II-mediated gene expression [2]. Interestingly, a similar pathogenic mechanism involving a point mutation within an intronic Alu element that creates aberrant splicing has been described in ornithine aminotransferase deficiency [23].

The homozygous intronic variant of *CTDP1* responsible for CCFDN syndrome is embedded within a distinct haplotype background due to a strong founder effect in the Roma ethnic group. Morar et al. conducted a comprehensive genetic study of the Roma population, examining multiple pathogenic variants, including the *CTDP1* c.863+389C>T variant [4]. The variant showed a strong founder effect among the Roma population, having a high carrier rate (~7% carrier rate). The disease-causing variant and its associated haplotype were not found in the 832 controls of non-Vlax Roma or other European populations included in the study, reflecting the genetic isolation and inbreeding practices that preserve this disease-causing variant within the Vlax Roma subgroup. Nonetheless, the Roma were described as a founder population composed of several subisolates, due to the significant differences in variant frequencies, haplotype divergence, and limited haplotype sharing, all suggesting internal heterogeneity. Microsatellite marker analysis around *CTDP1* showed that all affected chromosomes from the Vlax Roma participants share an identical haplotype of approximately 320 kb in length, supporting the conclusion that the disease-causing variant originated from a single ancestral event. The linkage disequilibrium surrounding the c.863+389C>T variant ensures that it can be reliably identified through nearby associated markers. This supports the hypothesis of a relatively recent disease-causing variant origin, estimated to have occurred 16–25 generations ago, before the divergence of the Vlax and Balkan Roma subpopulations [4]. This genetic structure highlights both the prevalence of CCFDN in certain Roma communities and the absence of the disease-causing variant in others, emphasizing the importance of understanding population-specific linkage patterns for genetic mapping of disease-associated genes and for efficient carrier screening.

On the other hand, in 2014 a population-based study screened 427 Croatian Bayash Roma individuals from two regions (Baranja and Međimurje) for variants causing several rare disorders, including the founder *CTDP1* disease-causing variant responsible for CCFDN syndrome [24]. While a carrier rate of 1.5% was found for the hereditary motor and sensory neuropathy type Lom disease-causing variant in Baranja, no carriers of the CCFDN causing variant were detected among 250 individuals tested across both populations. This absence contrasts with a reported ~2.6% carrier rate for CCFDN in broader Vlax Roma groups. The study highlights substantial differences in genetic disease variant frequencies between geographically and linguistically distinct Roma subgroups, likely reflecting varied migration histories, endogamy, and genetic drift [24]. These findings highlight how essential it is to understand the specific genetic background of Roma subgroups to provide accurate carrier screening and better support for individuals and families affected by these conditions.

## 4. Phenotypic Spectrum of CCFDN Syndrome

### 4.1. Clinical Features of CCFDN Syndrome

CCFDN is defined by a complex phenotype which involves several organs and systems [25]. Despite its complex phenotype, some specific features provide a relatively quick clinical diagnosis [26]. The syndrome is primarily characterized by bilateral congenital cataracts, which often requires early surgical intervention, and can be accompanied by microcornea, microphthalmos, and micropupils [2]. Additional ocular features include floppy eyelids, dense eyelashes, nystagmus, and strabismus [27].

Early motor and intellectual development are delayed, with most patients starting to walk between 2 and 3 years and speaking after 3 years [28].

Neurologically, a symmetric, distal, predominantly motor peripheral neuropathy is the hallmark feature. Motor impairment begins in infancy and worsens progressively, causing severe disability by the third decade. Tendon reflexes are diminished or absent, initially in the lower limbs [28]. The peripheral neuropathy in CCFDN primarily affects distal motor fibers, with electrophysiological studies showing uniform conduction slowing in the demyelinating range and relatively preserved sensory action potentials. Nerve biopsies reveal primary hypomyelination affecting both large and small fibers, alongside progressive demyelination/remyelination and, in older patients, axonal degeneration. Schwann cells fail to produce myelin of appropriate thickness relative to axon diameter. The exact cause of this hypomyelination remains unclear: it may result from an abnormal axonal signal, a Schwann cell response failure to normal axonal cues, or a primary defect in Schwann cells after initial axonal signaling. Ultrastructural studies have not definitively distinguished between these possibilities, reflecting the complexity of myelination regulation in CCFDN [25,29]. Other neurological findings include bilateral extensor plantar responses, mild chorea, ataxia, upper limb tremor, and frequent abnormal electroencephalographic slowing. CNS imaging shows age-related diffuse cerebral and spinal cord atrophy, including lateral ventricle enlargement and involvement of the medulla oblongata; diffusion tensor imaging further suggests axonal loss in the cerebellar vermis and brainstem regions [25].

Facial dysmorphism is a consistent feature, including a prominent nasal bridge, thickened lips, and micrognathia, contributing to a recognizable phenotype [22]. Skeletal abnormalities such as pes cavus or pes equinovarus with clawed toes, claw hand deformities, and kyphoscoliosis are common, often leading to reduced respiratory capacity [28].

Endocrine afflictions, notably hypogonadotropic hypogonadism, short stature, and delayed puberty, are frequently reported [2]. Endocrine abnormalities encompass decreased sex hormone levels with normal secondary sexual characteristics but frequent irregular menstrual cycles and secondary amenorrhea in adult females. Reduced bone mineral density affects both cortical and trabecular bone compartments [28].

Additional clinical concerns include episodes of severe myalgia and rhabdomyolysis, likely para-infectious in nature, posing risks for acute kidney injury [28]. Importantly, patients with CCFDN have an increased risk of anesthesia-related complications, including rhabdomyolysis and pulmonary edema, requiring careful perioperative management [2].

**Figure 4:**
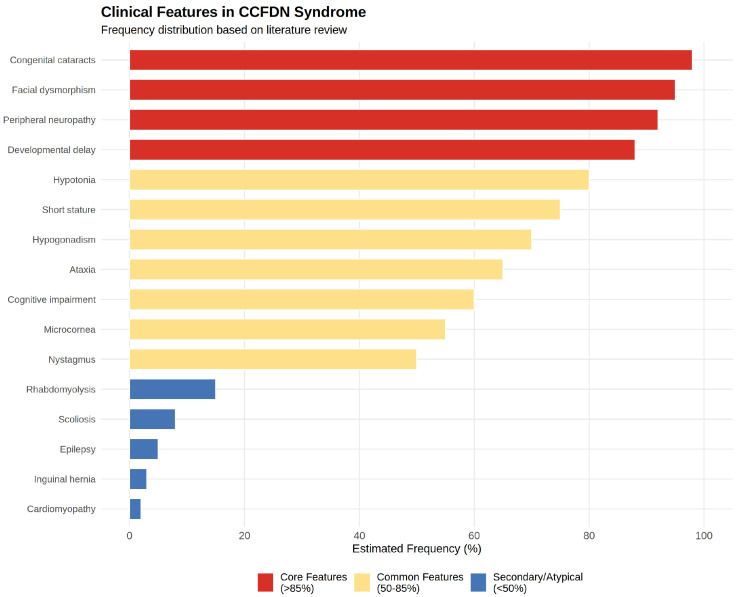
Estimated frequency distribution of phenotypic features in CCFDN patients based on literature review.

Table 1 (see below) summarizes key clinical and genetic studies describing the phenotypic spectrum and patient demographics of CCFDN syndrome across diverse Roma populations. Although not a systematic review, the table integrates 17 comprehensive studies from observational cohorts, genetic screenings, and case reports, providing a consolidated resource to appreciate the multi-systemic impact and phenotypic spectrum of CCFDN syndrome across diverse populations. In addition to patient ages, ethnic origins, and main reported features, we included a dedicated column highlighting key findings and contributions of each paper to facilitate a clear understanding of the evolving knowledge in the field. This overview underscores the importance of early molecular diagnosis and tailored multidisciplinary care for affected individuals. To facilitate comprehension of the complex phenotypic spectrum, Figure 4 provides a visual summary categorizing clinical features by estimated frequency into core features (>85% of patients), common features (50–85%), and secondary/atypical features (<50%), based on manual analysis of the reported cases across all studies. The figure was generated using R version 4.3.1 with ggplot2 and dplyr packages.

### 4.2. Differential Diagnosis and Overlapping Neurodevelopmental Disorders

When considering CCFDN syndrome, it is essential to distinguish it from other rare syndromes with overlapping clinical features, particularly Marinesco-Sjögren syndrome (MSS). Both disorders may present with intellectual impairment, congenital cataracts, and peripheral neuropathy. However, certain clinical features come to aid in the differential diagnosis of CCFDN and MSS. CCFDN is characterized by striking facial dysmorphism and a markedly severe hypomyelinating neuropathy, features which are not typical for MSS. In contrast, MSS is defined by the presence of chronic myopathy and the necessity of cerebellar involvement for diagnosis, both of which are not primary characteristics of CCFDN [30]. The genetic locus associated with MSS has been mapped to chromosome 5q31with mutations in the *SIL1* gene identified as the causative factor [40]. Even though there has been speculation regarding a connection between these two syndromes [41], genetic testing has not identified *CTDP1* disease-causing variants in individuals with MSS [2].

In addition, studies have expanded the phenotypic spectrum of *CTDP1* causing variants to include contributions to Charcot-Marie-Tooth disease (CMT). While *CTDP1* disease-causing variant is classically known as the genetic basis of CCFDN, genetic epidemiology studies in neuropathy cohorts have identified *CTDP1* disease-causing variants in a small subset of patients diagnosed with CMT, particularly within demyelinating CMT1 subtypes. For instance, a genetic screening study identified *CTDP1* accounting for 0.8% from the total of 59.9% disease-causing genes, highlighting that *CTDP1* is among the rarer but relevant genetic contributors to hereditary neuropathies [42]. This finding was also suggested in a review that included *CTDP1* as one of the genes responsible for autosomal recessive forms of CMT [43]. Clinically and electrophysiologically, these cases exhibit typical demyelinating features such as slowed nerve conduction velocities and myelin abnormalities. This highlights the relevance of *CTDP1* in genetic diagnostic panels for demyelinating hereditary neuropathies with overlapping clinical presentations to ensure comprehensive molecular characterization.

Additional considerations in differential diagnosis include rare neurodevelopmental disorders caused by pathogenic or likely pathogenic variants in genes functionally interacting with *CTDP1*, such as *INTS1* and *INTS8*. A recent report of two Chinese siblings with biallelic *INTS1* disease-causing variant revealed a neurodevelopmental syndrome sharing overlapping clinical features with CCFDN, including intellectual disability, cataracts, facial dysmorphism, motor impairments, and skeletal abnormalities, but notably lacking the peripheral neuropathy typical of CCFDN. Genetic interaction network analyses demonstrated that *INTS1*, *INTS8*, and *CTDP1* participate in the same molecular pathway regulating RNA polymerase II function, explaining phenotypic intersections and distinctions [44]. This emerging evidence shows how important it is to look beyond a single gene and consider variants in other genes belonging to the same transcriptional complex, especially when patients present with overlapping clinical features. It also highlights the value of comprehensive genomic testing in uncovering these underlying causes. The absence of neuropathy in patients with *INTS1*-related disorders, together with the severe cognitive impairment that differs from CCFDN, may assist in clinical differentiation, refining diagnosis and informing targeted genetic counselling.

## 5. Overview of Potential Therapeutic Approaches to *CTDP1*-Related Disorders

Finding effective treatments for CCFDN syndrome remains particularly challenging due to its rarity, being predominantly found in the Roma population and a few other groups, combined with its clinical heterogeneity, and a limited understanding of the underlying molecular mechanisms responsible for the disease. To address these challenges, we discuss here an overview of potential therapeutic alternatives for *CTDP1*-related disorders, focusing on emerging molecular and genetic approaches as well as strategies aiming to correct the fundamental splicing defects caused by the founder disease-causing variant.

### 5.1. Transcriptomics and Antisense Oligonucleotide (ASO) Drugs

Transcriptomic studies, such as bulk RNA sequencing, might offer a powerful approach for uncovering potential therapeutic targets and for guiding drug discovery. By comparing gene expression profiles between affected individuals and healthy controls, transcriptomics can reveal disease-specific dysregulated pathways, gene networks, and molecular signatures that drive pathology. Transcriptomic data can highlight genes which are either up-regulated or down-regulated in CCFDN, suggesting potential candidates for inhibitory or activatory targeting [45].

Data regarding gene expression is often used for drug repurposing, a valuable drug discovery approach that aims to identify new indications for known drugs [46]. Drug-induced human cell line transcriptomic data are used to identify potential pharmacological targets and pathways, as well as for anticipating new therapeutic indications for currently available medications [47]. Most of these methods use an inverse correlation approach, searching for medications that reverse the expression profile within a specific disease. For instance, the inverse correlation approach has been used to find medications that effectively treat colorectal cancer [48], prostate cancer [49], and inflammatory bowel disease [50]. In addition, gene knockdown and gene overexpression transcriptomic data are used for identifying correlations between disease-specific gene expression profiles and altered gene expression profiles linked to genetic defects. Namba et al. proposed a novel approach called target repositioning, which is a trans-disease method that integrates gene knockdown and gene overexpression signatures with disease specific gene transcriptomic signatures for therapeutic target prediction, by considering the similarities between diseases [46]. This method can differentiate between inhibitory and activatory targets, while also predicting targets for orphan proteins without known relationships.

Moreover, transcriptomics can identify splicing defects and aberrant isoforms, which might be amenable to splice-correcting therapies [51]. This could be particularly relevant in CCFDN, since the founder intronic disease-causing variant results in aberrant splicing. Antisense oligonucleotides (ASOs) might be a promising therapeutic strategy aiming to mask the cryptic splice site or to restore normal splicing patterns. ASOs are short, synthetic nucleic acid sequences designed to selectively bind to pre-mRNA near the mutant intronic region, thereby blocking the access of the spliceosome to aberrant splice sites, while also promoting the use of canonical splice junctions. Nusinersen is an example of an antisense oligonucleotide drug, approved by the U.S. Food and Drug Administration (FDA) in 2016 for treating spinal muscular dystrophy, by binding to an intronic sequence in the SMN2 gene, which leads to the inclusion of exon 7 and to an increase in the production of functional survival motor neuron protein [52,53]. ASOs modulate splicing primarily through a steric-blocking mechanism: they prevent binding of splicing factors to aberrant splice sites without degrading the target RNA (i.e., they typically have a fully modified backbone chemistry that precludes recruitment of RNaseH (Ribonuclease H)), preserving transcript levels which is critical for maintaining normal mRNA abundance. Chemical modifications to ASOs improve their stability, cellular uptake, binding affinity, and reduce off-target effects and toxicity, which are crucial for therapeutic efficacy [54]. Importantly, ASOs can increase levels of functional mRNA and protein by preventing generation of non-productive transcripts that are usually degraded via nonsense-mediated decay, thus boosting functional protein expression in a dose-dependent manner [55]. A major challenge in developing ASO therapies for CCFDN lies in effective delivery to relevant tissues, such as peripheral nerves and lens epithelial cells, which are mainly affected in this syndrome. Strategies for enhancing the tissue-specific uptake, improving stability, and minimizing off-target effects are critical for the clinical success of ASO-based interventions [56,57]. Such strategies include the chemical conjugation of splice switching ASOs to lipids, peptides or antibodies that enhance the extra-hepatic distribution of the drug [58,59]. A number of these approaches are already in clinical development, e.g., transferrin receptor type 1 (TfR1) antibody conjugates, developed by Avidity Biosciences and Dyne Therapeutics [60,61]. Taken together, the specific splicing defect caused by the founder *CTDP1* disease-causing variant in CCFDN makes antisense oligonucleotides a rational and potentially effective therapeutic alternative, mirroring successes seen in other rare spliceopathies. However, overcoming the pharmacokinetic and delivery challenges remains critical before clinical application [62].

### 5.2. Small Molecule Splicing Modulators

An emerging strategy for correcting aberrant splicing in genetic diseases involves the use of small molecule splicing modulators. Unlike antisense oligonucleotides, these compounds offer the advantage of oral bioavailability and systemic distribution, making them particularly attractive for clinical use in disorders which affect multiple tissues. Small molecule modulators can alter spliceosome activity by binding to spliceosomal proteins, such as SF3B1, or by stabilizing the interactions between the spliceosome and pre-mRNA at critical splice sites. By influencing the splicing outcome, this mechanism highlights the therapeutic potential of directly targeting the splicing machinery [63]. Risdiplam is the first FDA-approved small molecule splicing modulator for the treatment of spinal muscular atrophy (SMA), introduced in 2020 [64]. This molecule stabilizes the interaction between the U1 snRNP and the 5′ splice site of exon 7, compensating for a weak splice site and restoring proper splicing. The inclusion of exon 7 in the SMN2 transcript promotes the production of functional SMN protein [65]. Notably, *CTDP1* serves as a molecular bridge linking transcription elongation to RNA splicing through its interactions with key spliceosomal components, including MEP50, which plays a role in the assembly of spliceosomal snRNPs [15]. This functional link between transcription and splicing suggests that the pharmacological modulation of spliceosome components could have dual effects, namely enhancing splicing fidelity while simultaneously influencing transcriptional regulation. Such a multifaceted approach could be impactful for correcting complex gene expression defects responsible for severe diseases, such as CCFDN. However, the design of small molecules that can specifically modulate splicing is challenging due to the complex nature of RNA structures and the spliceosome, but advances in structural biology are broadening the range of potential candidates [66].

### 5.3. Gene Replacement Therapy

Gene replacement therapy represents a promising therapeutic approach for CCFDN syndrome by delivering a functional copy of the *CTDP1* gene to affected cells. Given that the *CTDP1* complementary DNA (cDNA) is approximately 3.5 kb in length, it might be compatible with packaging into adeno-associated virus vectors (AAV), which are widely used for in vivo gene delivery due to their ability to transduce post-mitotic cells. However, recent safety concerns have emerged regarding systemic AAV administration, as high viral particle doses required for widespread tissue targeting have led to serious adverse events in some clinical trials [67]. Therefore, careful assessment of delivery routes, dosing, and immune responses will be critical before considering such an approach for CCFDN. Lentiviral vectors remain an alternative, especially for ex vivo gene therapy approaches. However, achieving efficient and tissue-specific delivery remains a significant challenge, as CCFDN affects multiple tissues, including peripheral nerves and crystalline lenses. Moreover, the critical role of *CTDP1* in transcription regulation raises concerns regarding the potential risks of overexpression, which could disrupt normal cellular processes. Careful control of transgene expression levels will be essential for avoiding adverse effects. Other gene replacement studies in inherited peripheral neuropathies, such as CMTX1, where lentiviral delivery of wild-type GJB1 showed therapeutic benefit, have underlined the potential of this technique, but have also highlighted the complexity of translating these strategies to clinical use [68].

### 5.4. Genome and Transcriptome Editing: CRISPR-Cas and ADAR Approaches

Genome editing using CRISPR-Cas technology involves a guide RNA (gRNA) that directs the Cas nuclease to a specific DNA sequence, where Cas9 creates a precise double-stranded break (DSB) near a protospacer adjacent motif (PAM). Following cleavage, the cell repairs the DSB via two main pathways: non-homologous end joining (NHEJ) and homology-directed repair (HDR). NHEJ is an error-prone mechanism that directly ligates DNA ends, often introducing insertions or deletions (indels) which can disrupt gene function. In contrast, HDR uses a homologous DNA template to accurately restore or modify the target sequence, enabling precise gene correction or insertion [69]. Importantly, HDR is restricted to the late S and G2 phases of the cell cycle, because it depends on the presence of sister chromatids as templates for repair, which are available only during DNA replication [70]. Conversely, NHEJ is active throughout the entire cell cycle, including non-dividing or quiescent cells [71]. This means that cells which are post-mitotic or have limited cell division, such as neurons and certain peripheral nerve cells affected in CCFDN syndrome, may not support efficient HDR-based genome editing [72]. Moreover, HDR efficiency can potentially be improved by manipulating the cell cycle to increase the proportion of cells in HDR-permissive phases, thus enhancing precise genome editing outcomes [73,74,75]. Therefore, therapeutic strategies using CRISPR-Cas for disorders involving such cells must carefully consider this limitation, possibly favoring NHEJ-based approaches or developing alternative precision editing methods compatible with non-dividing cells.

Two advanced CRISPR-based editing methods are base editing and prime editing. Base editing enables precise conversion of one DNA base into another (for example, cytosine to thymine, or adenine to guanine), without inducing double-stranded DNA breaks (DSBs), by using a fusion of catalytically impaired CRISPR-associated protein 9 (Cas9) and a deaminase enzyme. However, base editing is limited to four types of base substitutions and may cause off-target or bystander edits [76]. Prime editing, a newer technique, expands editing capabilities by combining a Cas9 nickase fused to a reverse transcriptase with a prime editing guide RNA (pegRNA) that both directs target binding and encodes the desired edit. This system nicks only one DNA strand and uses reverse transcription to install all twelve possible base substitutions, as well as small insertions and deletions, with high precision and fewer off-target effects. Because prime editing does not generate DSBs, it reduces risks of chromosomal rearrangements and cellular toxicity associated with traditional CRISPR-Cas9 editing [76]. Despite the advantages, genome editing therapies face significant challenges and safety concerns, including unintended off-target modifications (such as deletions, disease-causing variants, or mosaicism with unknown consequences) and immune responses to editing proteins [77].

Adenosine Deaminase Acting on RNA (ADAR)-mediated RNA editing is an emerging therapeutic approach that enables precise correction of pathogenic variants at the RNA level, without altering the genome. ADAR enzymes can facilitate the deamination of adenosine to inosine (A-to-I) in double-stranded RNA. During translation, inosine is recognized as guanosine, hence enabling the correction of mutant bases in transcripts [78]. This type of editing can correct certain point disease-causing variants at the RNA level and can modulate splicing by targeting adenosine in pre-mRNA. However, since the CCFDN causing variant is a cytosine-to-thymine substitution in an intronic region, ADAR enzymes cannot directly revert this change because they do not catalyze cytosine or thymine base conversions. Nevertheless, recent studies show that ADAR editing can influence splicing by editing adenosines near splice sites or regulatory elements, indirectly modulating aberrant splicing. For example, Schneider et al. demonstrated that ADAR-based site-directed RNA editing (SDRE) might be able to target deep intronic variants, which affect splicing, by carefully designed guide RNAs that recruit ADAR to pre-mRNA, sometimes combining RNA editing with antisense oligonucleotide-like effects, in order to restore normal splicing patterns [79]. This suggests a potential, but indirect, therapeutic avenue for CCFDN by targeting the RNA elements involved in splice site recognition or splicing enhancers near the disease-causing variant.

Recent studies have revealed that ADAR1, traditionally recognized for its adenosine-to-inosine RNA editing activity, also regulates alternative splicing through RNA editing-independent mechanisms. ADAR1 can bind to double-stranded RNA, interacting directly with spliceosome components and splicing regulators, therefore being able to exert an influence on splice site selection without catalyzing nucleotide changes. This editing-independent role extends to modulation of microRNA biogenesis and innate immune responses, by affecting RNA processing and surveillance pathways. These multifunctional activities position ADAR1 as a critical coordinator of RNA metabolism beyond its enzymatic editing function. Understanding the dual role of ADAR1 is particularly relevant for disorders such as CCFDN, where disruptions in RNA processing and splicing contribute to the physiopathological pathway. Consequently, targeting both RNA-protein interactions and the splicing machinery involved in these processes may offer new therapeutic opportunities for such conditions [80].

Genome and transcriptome editing technologies represent significant advances in molecular medicine. However, their clinical translation remains in the early experimental stage. Optimization is required to ensure long-term safety, efficacy, and precision in human tissues. Efficient delivery remains a critical challenge, as most strategies rely on viral vectors such as AAV, which pose additional risks, particularly for systemic administration [81,82]. While these approaches hold considerable promise for future CCFDN therapies, extensive preclinical studies are needed to develop safe and targeted delivery systems prior to clinical implementation.

### 5.5. Other Potential Therapeutic Approaches

An alternative therapeutic strategy for CCFDN could involve boosting the expression of the wild-type *CTDP1* allele for increasing the proportion of functional full-length protein. This approach is conceptually appealing, especially since studies indicate that in CCFDN patients, approximately 30% of CTDP1 protein is normal and full-length, while 70% is aberrant and likely non-functional due to aberrant splicing [2]. One promising method to achieve this enhancement is through the use of antisense oligonucleotides (ASOs) targeting regulatory regions in the 5′ untranslated region (5′UTR) of the *CTDP1* mRNA, such as upstream open reading frames (uORFs), which typically inhibit translation. Some studies have demonstrated that steric blockade of uORFs or other inhibitory 5′UTR elements by ASOs can effectively increase translation initiation and elevate protein levels, with increases ranging from 30% to 150% in various models [83]. Liang et al. [84] demonstrated that ASOs can enhance translation by sterically blocking not only upstream open reading frames (uORFs) but also other inhibitory secondary structures in 5′UTRs, thereby increasing protein expression in cells and in vivo models. On the other hand, Winkelsas et al. [85] applied ASOs targeting the 5′ UTR of SMN2 to increase SMN protein levels primarily by stabilizing SMN2 mRNA rather than by inhibiting uORF-mediated repression. More recent work reveals that 5′UTR secondary structures, particularly double-stranded RNA elements adjacent to uORFs, regulate translation by modulating uORF initiation, and that ASOs can be designed to precisely tune this interplay to either enhance or repress protein expression [86]. Together, these approaches highlight the potential of ASO-based therapies to modulate *CTDP1* expression at multiple regulatory levels, including translation initiation and mRNA stability, offering promising avenues for restoring functional protein and mitigating CCFDN pathology.

## 6. Conclusions

CCFDN syndrome, caused by a unique intronic founder variant in the *CTDP1* gene predominantly affecting the Roma population, exemplifies a rare but clinically significant spliceopathy with multisystem involvement. *CTDP1* plays a crucial role in transcription regulation, RNA splicing, and genome integrity, and its dysfunction leads to the complex phenotype characterized by congenital cataracts, facial dysmorphism, and progressive neuropathy. Advances in molecular understanding have highlighted *CTDP1*’s diverse biological functions, its involvement in DNA repair and cancer biology, and its emerging role as an autoimmune biomarker. Therapeutic approaches remain challenging; however, potential avenues such as ASOs, small molecule splicing modulators, gene replacement therapy, DNA and RNA editing hold potential for targeted intervention. Among these, ASOs represent a particularly versatile and adaptable strategy, with the ability to modulate *CTDP1* expression at multiple regulatory levels. Continued research integrating transcriptomic and functional studies is essential to unravel the full pathogenic mechanisms and to develop effective, precise treatments for *CTDP1*-related disorders. Future investigations should also explore potential genetic modifiers and environmental factors that may contribute to the observed phenotypic variability among CCFDN patients carrying the same founder mutation.

## Figures and Tables

**Table 1:** Key studies describing phenotypic aspects and patient demographics of CCFDN syndrome across diverse Roma populations. (Abbreviations: No. = number of patients; OA = original article; CR = case report).

Author	Year	Article Type	No.	Age (Years)	Origin	Reported Phenotype/Main Findings	Paper Highlights
Tournev et al. [25]	1999	OA	50	Infants–44	Balkan Roma	Congenital cataracts, microcorneas, lower and upper limb motor neuropathy, moderate nonprogressive cognitive deficit, pyramidal signs and mild chorea, short stature, characteristic facial dysmorphism, hypogonadotrophic hypogonadism	Early electro-physiological characterization
Tournev et al. [29]	1999	OA	4	4–32	Bulgaria	Peripheral neuropathy, diffuse hypomyelination, reduced nerve conduction velocity with relative preservation of sensory action potentials, progressive demyelination/remyelination and axonal degeneration for adults	A developmental hypomyelination process with superimposed degenerative changes over time
Varon et al. [2]	2003	OA	85	4–47	Vlax Roma	Congenital cataracts, facial dysmorphism, demyelinating neuropathy, short stature, hypogonadism, mild cognitive impairment, cerebellar signs; The disease-causing variant determines aberrant splicing with insertion of Alu element in *CTDP1* mRNA, disrupting FCP1 phosphatase function	Genetic homogeneity and founder disease-causing variant in the Vlax Roma population with an inferred variant origin within ~16 generations; CCFDN is caused by a rare aberrant splicing mechanism involving an Alu insertion
Müllner-Eidenböck et al. [27]	2004	OA	9	1.3–16.8	Eastern part of Serbia, close to the border with Romania	Peripheral, demyelinating neuropathy, ataxia, muscular atrophy, facial dysmorphism, bilateral congenital cataracts, microcornea, microphthalmos, and micropupil, floppy eyelid syndrome, pseudoptosis, nystagmus and congenital esotropia, visual impairment	Increased inflammatory reaction to contact lenses and intraocular lenses
Shabo et al. [30]	2005	CR	1	5	Hungarian Roma	Bilateral congenital cataract, normal intellect, motor development delay, congenital hip dysplasia, progressive pes equinovarus adductus, demyelinating neuropathy, reduced conduction velocities	Orthopedic deformities reported, no onion bulbs or Schwann cell inclusions were observed at sural nerve biopsy
Mastroyianni et al. [31]	2007	CR	1	3	Greek Roma	Congenital cataracts, hypotonia, absent tendon reflexes, pesequinovarus, paretic flexion position of the hands, recurrent infectious rhabdomyolysis	Anti-influenza Vaccination recommendation
Iagaru et al. [32]	2008	CR	1	9	Romanian Roma	Congenital cataracts, mild ataxia, hypotonia, acute rhabdomyolysis, somatic and mental retardation	Initially considered as a possible case of MSS
Cordelli et al. [33]	2010	CR	1	10	Roma (mother from Bosnia and father from Serbia)	Bilateral congenital cataracts, hypotonia, motor and speech delay, progressive demyelinating motor and sensory polyneuropathy, mild facial abnormalities; CNS changes reveal a likely process of deficient or damaged myelin (hypomyelination and demyelination) paralleling peripheral nerve pathology; Diffusion Tensor Imaging detected microstructural damage of white matter and involvement of the basal ganglia; Magnetic Resonance Spectroscopy indicates preserved neurons but altered glial cell	First longitudinal study demonstrating progressive involvement of cerebral white matter; Supports hypothesis that the disease-causing variant in *CTDP1* disrupts transcription regulation affecting both central and peripheral nervous system myelin integrity; Emphasizes importance of clinical and neuroimaging follow-up
Tzifi et al. [34]	2011	CR	1	Early infancy	Greek Roma	Variable clinical presentation; not all diagnostic criteria need to be present early to suspect disease, especially in high-risk population; Clinical features, especially motor delay and peripheral neuropathy, evolve slowly but progression occurs into severe disability in adulthood	Demonstrates utility of molecular genetic testing for definitive diagnosis and prenatal counseling
Petra Lassuthova et al. [22]	2014	OA	10	3–18	Czech Roma	Bilateral congenital cataract, microphthalmos, early cataract surgery, delayed motor milestones, paleocerebellar gait, mild mental retardation; Ophthalmological complications include dense bilateral cataracts, microphthalmos, nystagmus, and secondary glaucoma	Largest pediatric cohort with CCFDN outside Bulgaria; Highlights need for lifelong ophthalmologic and neurologic monitoring to manage progressive complications
Walter et al. [35]	2014	OA	10	1–30	Balkan and Central Europe Roma	Presenting features: bilateral congenital cataracts, strabismus, facial dysmorphism, short stature, and demyelinating sensory-motor neuropathy; Progressive worsening of distal muscle weakness (small hand muscles, foot extensors); ataxia scores remained stable or improved over time; Sensory nerve conduction velocities slowed initially with normal amplitudes but later showed reduced amplitudes indicating axonal loss in sensory and motor nerves; Vitamin deficiencies (notably vitamin E and D) were observed in some patients; Brain MRI showed no cerebellar atrophy but unspecific changes like accentuated ventricles	Provides the first detailed 10-year longitudinal clinical and paraclinical follow-up in CCFDN, illustrating its progressive disabling course; Shows benefit of early and ongoing physiotherapy in improving ataxia and maintaining function; Suggests potential role for liposoluble vitamin supplementation
Chamova et al. [36]	2015	OA	22	4–47	Bulgaria	Mild intellectual deficit, and borderline intelligence; Brain MRI: diffuse cerebral atrophy, lateral ventricle enlargement, and localized lesions in the subcortical white matter, varying in size and quantity; more severe demyelination in older patients	When compared to the healthy control group, CCFDN patients scored significantly lower on all psychometric tests that assessed several cognitive domains. Only the impairment of short-term verbal memory was shown to be statistically significantly correlated with the MRI changes in the correlation study of structural brain changes and cognitive impairment
Makrygianni et al. [3]	2017	CR	2	5	Greek Roma	Report of two siblings showing pronounced intrafamilial variability including a novel clinical feature: cardiomyopathy in the brother, not previously documented in CCFDN; One sibling had microcephaly, dystonia, ataxia, and sensorimotor demyelinating neuropathy with axonal loss; The deceased brother had acquired microcephaly, developmental delay, and fatal cardiorespiratory collapse due to severe dilated cardiomyopathy triggered by febrile illness	Expands the clinical spectrum of CCFDN syndrome to include cardiomyopathy and acquired microcephaly, underscoring variable expressivity; Highlights heat shock protein dysregulation as a potential pathogenic mechanism for rhabdomyolysis and cardiac complications
Masters et al. [37]	2017	CR	1	9	Romanian Roma	Initially misdiagnosed case with Guillan Bare syndrome; apart from classical clinical features of CCFDN, the case presented with tibialis tendon transfer and bilateral orchidopexy	Clarifies that CCFDN syndrome patients are at high risk for rhabdomyolysis with certain anesthetic agents but should not be labeled as malignant hyperthermia susceptible; Suggests avoidance of depolarising muscle relaxants, caution use of nondepolarising muscle relaxants, and minimal exposure to volatile anaesthetics.
Jurca et al. [26]	2018	CR	2	11, 13	Romanian Roma	Two siblings diagnosed with CCFDN. Both siblings exhibit the pathognomonic triad of CCFDN. The boy experienced a life-threatening episode of parainfectious rhabdomyolysis. Additional phenotypic features included congenital right inguinal hernia in both siblings. The girl also presented epilepsy with generalized tonic–clonic seizures responsive to usual antiepileptic drugs	Identification of a novel possible phenotype feature (inguinal hernia) in CCFDN
Dudakova et al. [38]	2020	CR	1	41	Czech Roma	Severe peripheral polyneuropathy, history of bilateral congenital cataracts, microcornea, and horizontal nystagmus, intellectual disability, facial dysmorphism, delayed psychomotor development; delayed definitive diagnosis, which was not made until 41 years [39]	Demonstrates the coexistence of two distinct monogenic ocular disorders (nanophthalmos and CCFDN) segregating within one consanguineous Roma family; Stresses the need for increased awareness of rare founder diseases in minority populations to improve timely diagnosis and care
Hudec et al. [39]	2022	CR	1	13	Czech Roma	Case presenting with extensive posterior neuromuscular scoliosis (Cobb angle 83°) that underwent corrective surgery	First documented successful endotracheal intubation in a 13-year-old patient with CCFDN syndrome undergoing major orthopedic surgery; Demonstrates the safety and efficacy of total intravenous anesthesia protocols with rocuronium and sugammadex

## Data Availability

No new data were created or analyzed in this study. Data sharing is not applicable to this article.

## Abbreviations

aa	amino acids
ADAR	Adenosine Deaminase Acting on RNA
ASOs	Antisense oligonucleotides
BRCT	BRCA1 C-terminal
Cas9	CRISPR-associated protein 9
CCFDN	Congenital Cataracts, Facial Dysmorphism, and Neuropathy
cDNA	complementary DNA
CK2	casein kinase 2
CRISPR	Clustered Regularly Interspaced Short Palindromic Repeats
CTD	Carboxy-Terminal Domain
CTDP1	Carboxy-Terminal Domain phosphatase subunit 1
CUL3	E3 ubiquitin ligase Cullin
DSBs	double-stranded DNA breaks (DSBs)
FCP1	F-Cell Production 1
FCPH	FCP homology
HAD	haloacid dehalogenase
MEFs	mouse embryonic fibroblasts
mRNA	microRNA
pegRNA	prime editing guide RNA
Refs	References
rRNA	ribosomal RNA
SDRE	site-directed RNA editing
siRNA	small interfering RNA
SINEs	short interspersed nuclear elements
SMA	spinal muscular atrophy
snRNPs	small nuclear ribonucleoproteins
TIF-IA	transcription initiation factor IA
